# LGMD D2 TNPO3-Related: From Clinical Spectrum to Pathogenetic Mechanism

**DOI:** 10.3389/fneur.2022.840683

**Published:** 2022-03-04

**Authors:** Roberta Costa, Maria Teresa Rodia, Serafina Pacilio, Corrado Angelini, Giovanna Cenacchi

**Affiliations:** ^1^Department of Biomedical and Neuromotor Sciences–DIBINEM, Alma Mater Studiorum University of Bologna, Bologna, Italy; ^2^Applied Biomedical Research Center–CRBA, IRCCS St. Orsola Hospital, Alma Mater Studiorum University of Bologna, Bologna, Italy; ^3^Laboratory for Neuromuscular Diseases, Campus Pietro d'Abano, University of Padova, Padova, Italy

**Keywords:** muscle biopsy, LGMD D2, transportin-3, TNPO3, atrophy, LGMD, HIV, nucleus

## Abstract

Limb-girdle muscular dystrophies (LGMDs) are clinically and genetically heterogeneous diseases presenting with a wide clinical spectrum. Autosomal dominant LGMDs represent about 10–15% of LGMDs and include disorders due to defects of DNAJB6, transportin-3 (TNPO3), HNRNPDL, Calpain-3 (CAPN3), and Bethlem myopathy. This review article aims to describe the clinical spectrum of LGMD D2 TNPO3-related, a rare disease due to heterozygous mutation in the *TNPO3* gene. *TNPO3* encodes for transportin-3, which belongs to the importin beta family and transports into the nucleus serine/arginine-rich (SR) proteins, such as splicing factors, and HIV-1 proteins, thus contributing to viral infection. The purpose of this review is to present and compare the clinical features and the genetic and histopathological findings described in LGMD D2, performing a comparative analytical description of all the families and sporadic cases identified. Even if the causative gene and mutations of this disease have been identified, the pathogenic mechanisms are still an open issue; therefore, we will present an overview of the hypotheses that explain the pathology of LGMD D2 TNPO3-related.

## Introduction

Limb-girdle muscular dystrophies (LGMDs) are a group of muscular diseases characterized by predominant proximal muscle weakness. Phenotypically, LGMD subtypes are highly variable in terms of age of onset, speed of disease progression, and overall severity; at a histopathological level, their main common feature is progressive muscle degeneration, although they do not share a common pathological mechanism (1). The estimated incidence for all forms is 1:100,000 and they are divided into two major subgroups: autosomal dominant forms (LGMD type D, according to the new classification) and autosomal recessive (LGMD type R). The new classification and definition of LGMDs also take into account age at onset, motor function, creatine kinase (CK) levels, muscle histology, and imaging (2). Moreover, recent advances in genome sequencing techniques have led to the pathological identification of 30 different LGMD genetic subtypes (3).

One subtype of dominant LGMD is caused by a mutation in the *TNPO3* gene (LGMD D2), which encodes for transportin-3 (TNPO3), a protein belonging to the importin beta-superfamily. TNPO3 normally mediates the nuclear import of Ser/Arg-rich (SR) proteins, a group of proteins involved in mRNA metabolism and splicing (4).

TNPO3-related LGMD D2 is a rare disease, first identified in an Italo-Spanish family (5–8); however, new affected families have recently been found in Europe (1, 9, 10) and sporadic cases have also been described (11, 12). The purpose of this review article is to present an overview of LGMD D2 based on the available literature, comparing the described clinical and MRI features and the genetic and histopathological findings, utilizing a thorough analysis of the original Spanish-Italian family (5, 6) and by performing a comparative analytical description of the new families and sporadic cases. In addition, we present some hypotheses on the pathogenetic mechanism of this disease which is still largely unknown.

## Clinical Features of LGMD D2 Families and Sporadic Cases

This LGMD disorder is widespread, mostly in the Caucasian population, with major geographical distribution in three areas of Spain (Valencia, Catalonia, and Murcia), with over 170 affected patients; an Italian branch of the Italo-Spanish kindred lives in North-East Italy. A new family in Hungary and one in Sweden have been described, and two sporadic Italian cases.

The first to be discovered was a large three-generation Italo-Spanish family, previously described in detail (5, 6) and the Italian branch, composed by an index case (a female now in her fourth decade of life) and her mother, showing a less severe phenotype with proximal weakness (13, 14). The LGMD D2 clinical phenotype shows high variability in the age of onset to the severity and progression of disease; the main common clinical features are represented by severe weakness, occurring first at onset in the pelvic lower girdle and later with disease progression in the axial and shoulder girdle, and marked atrophy of muscles. Cardiac involvement has not yet been described in LGMD D2 and in this respect, it can be considered an almost exclusively skeletal muscle expressed LGMD, aside from its bone manifestations, since arachnodactyly and scapular winging also characterize the phenotype. Phenotype severity does not always increase in successive generations and the clinical-genetic data available on the penetrance do not permit to definitively estimate it (15, 16).

**Figure 1:**
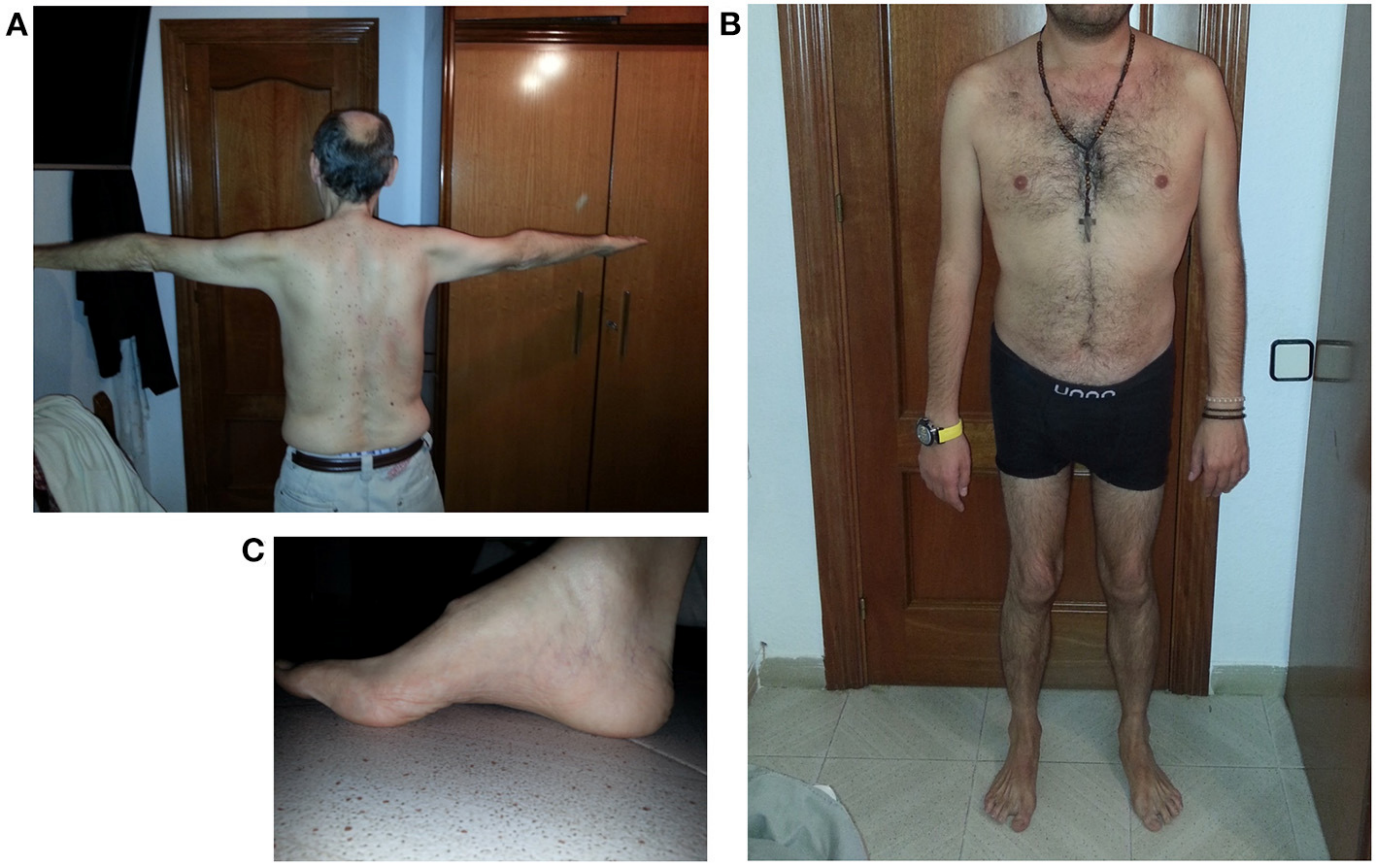
Clinical features of LGMD D2 cases. **(A)** Atrophy of upper girdle muscles, especially deltoid and triceps brachii; **(B)** lower girdle muscle atrophy; **(C)** pes cavus.

Figure 1 reports some clinical features of this LGMD D2 family.

A second family has been recently identified in Hungary and is composed of an index case, a woman, who was first examined at age 43, and her son (1, 10). Even in this family, the affected son had an earlier onset than his mother, who had decreased muscle bulk and finger and back muscle strength and difficulty swallowing from 40 years of age. Only one of her sons is affected, who started presenting signs of weakness in his first year of life. At present, he is 14 years and has a waddling gait, Gowers sign, he can climb stairs only when using a stick, and he also has signs of dysphagia with difficulty in swallowing.

A Swedish family with 3 patients representing subsequent generations (the proband, his mother, and his son) was described by Vihola et al. (9). All patients had delayed walking and generalized muscle atrophy and proximal weakness with a very early onset, but the disease remained stable until adulthood, when weakness slowly started to progress, differently from the Italo-Spanish family.

In addition to these families, two sporadic cases, with no familial history for neuromuscular disorders, have been described (11, 12). The first sporadic case identified had slowly progressive difficulty in walking and climbing stairs from the age of 35 and presented with proximal limb muscle atrophy with weakness of the shoulder girdle muscles without scapular winging. The symptoms progressively worsened and, almost 16 years after onset, he had severe shoulder and pelvic girdle muscle and proximal upper and lower limb muscle weakness, and also hand atrophy, and finger extensor weakness (11).

The second sporadic case was first examined at 4 years of age for global hypotonia and high CK, and at 10 years disclosed diffuse muscle hypotrophy and weakness. At the last neurological examination, at age 33, he had a waddling gait, weakness, and atrophy of limb-girdle muscles, and skeletal abnormalities like scapular winging, rigid spine, and scoliosis, as well (12). For both sporadic cases, next-generation sequencing (NGS) identified the same R818Q variant in *TNPO3* (7, 11, 12).

The phenotypic spectrum of LGMD D2 for both familial and sporadic cases is summarized in Table 1.

**Table 1:** Major clinical and histopathological features in the familial and sporadic cases of LGMD D2 TNPO3-related.

		**Number of patients presenting specific symptoms/alterations**						
		**Italo-Spanish**	**Hungarian**	**Swedish**	**Total**	**Total %**	**Sporadic**	**Total %**
Clinical features	Infancy/childhood onset (<15 years)	25/30	1/2	3/3	29	83	1/2	50
	Adult onset (>20 years)	8/30	1/2	0/3	9	26	1/2	50
	Early loss ambulation (<35 years)	3/30	0/2	0/3	3	9	0/2	0
	Scapular winging	4/30	1/2	0/3	5	14	1/2	50
	Skeletal abnormalities (long fingers, contractures of fingers flexor, pes cavus and scoliosis)	14/30	1/2	0/3	15	43	1/2	50
	Dysphagia	9/30	2/2	0/3	11	31	0/2	0
	Respiratory involvement	3/30	1/2	1/3	5	14	1/2	50
	Cardiac involvement	0/30	0/2	0/3	0	0	0/2	0
	Myopathic EMG	7/30	1/2	1/3	9	26	2/2	100
	Increased (mild elevation) CK level	18/30	0/2	1/3	19	54	2/2	100
Histopathologicial features	Fiber size variability	9/9	2/2	3/3	14	100	1/2	50
	Rimmed vacuoles	5/9	0/2	3/3	8	57	0/2	0
	COX-negative fibers	5/9	2/2	3/3	10	71	2/2	100
	Nuclear abnormalities	9/9	2/2	3/3	14	100	0/2	0
	Myofibrillar changes	9/9	0/2	3/3	12	86	2/2	100
	Fiber regeneration	0/9	0/2	0/3	0	0	0/2	0
	Autophagic vacuoles or myelinoid/membranous structures	5/9	1/2	3/3	9	64	0/2	0
	References	(8, 15)	(1, 10)	(9)			(11, 12)	

## Muscle MRI

**Figure 2:**
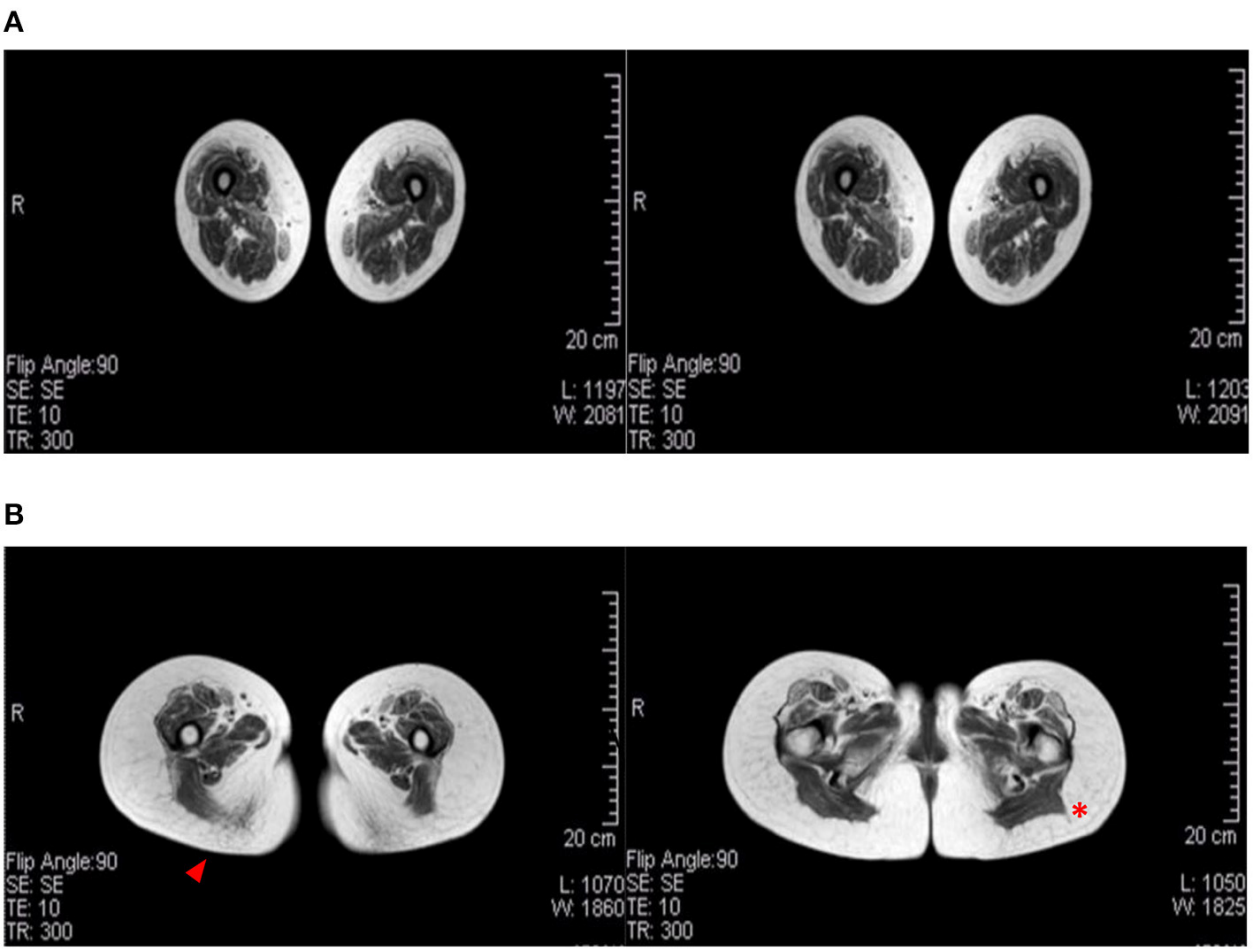
Muscular MRI radiological aspects. **(A)** A female patient (age 60) affected by LGMD D2 and mother of **(B)** the index female case of the Italian branch of the Italo-Spanish family (age 34). Advanced replacement and atrophy of the semitendinous (arrowhead) compared to the back muscles of the thigh of the wide side and the femoral rectus of the middle and small gluteus (red asterisk) to the illicit insertion.

Magnetic resonance imaging is used in LGMD to assess the severity and distribution of muscle involvement: in LGMD D2, a characteristic pattern of early and severe atrophy has been observed, especially in juvenile cases. In the Italo-Spanish kindred, muscle MRI showed variable involvement in scapulohumeral girdle muscles that appear better preserved than the pelvic-femoral and leg muscles (Figure 2). Fibro-fatty replacement also correlates with fiber atrophy and the intensity of MRI signal changes that appear in relationship with the degree of impairment and severity of clinical involvement, and the Mercuri scale is used to score scapular girdle, lumbar, thigh, and lower leg muscles (8, 14, 17).

Muscle MRI in the Swedish family showed severe diffuse and generalized fatty degenerative alterations in all pelvic and thigh muscles and slightly less severe but still diffuse changes in all the distal lower limb muscles. In one case, early diffuse degenerative changes were seen in all muscles (9).

In the Hungarian family, muscle MRI showed marked atrophy and fatty substitution in the pelvic girdle and anterior thigh muscles, while posterior muscles were relatively spared; posterior leg muscles showed marked atrophy and fatty replacement (1).

In the sporadic case, muscle MRI revealed a severe fibro-fatty substitution of bilateral anterior and posterior thigh muscles (11), an MRI pattern that is different from the first described family, where atrophy was the early prominent feature.

## Histopathological and Ultrastructural Analyses of Muscle Biopsy

Muscle biopsies showed heterogeneous histopathological features, in particular, chronic myopathic changes, increased variability of fiber size, central nuclei, fiber splitting, endo- and perimysial fibrosis, scattered degenerating fibers, and type 1 fiber prevalence. Common and peculiar aspects of muscle biopsies were diffuse and progressive muscle fiber atrophy, the presence of rimmed vacuoles, basophilic cytoplasmic regions, and enlarged nuclei with central pallor (8, 14). Immunohistochemical stains highlighted intracytoplasmic areas with the accumulation of cytoskeletal (desmin) and myofibrillar (myotilin) proteins, suggesting abnormalities in the intermyofibrillar network (14). Moreover, immunofluorescence for p62 demonstrated increased p62-positive aggregates in some atrophic fibers (18).

Electron microscopy confirmed fiber atrophy and showed autophagic vacuoles, abnormal mitochondria accumulations with rare paracrystalline inclusions, and many alterations of the myofibrillar component, with prominent disarray and accumulation of electron-dense material of possible Z-line derivation (5, 8, 18).

Histopathological findings similar to the ones previously described have been found in the muscles of patients from the Swedish and Hungarian families, and myofibrillar/cytoskeletal involvement was also confirmed by immunostaining for myotilin, desmin, tropomyosin, and alpha-actinin (9, 10, 19). Expression of p62 was also observed in the Swedish family, along with microtubule-associated proteins 1A/1B light chain 3, TDP-43, and ubiquitin. These last observations, together with p62 expression and the autophagic vacuoles described at ultrastructural levels, suggest induction of the autophagosome degradation pathway (9).

**Figure 3:**
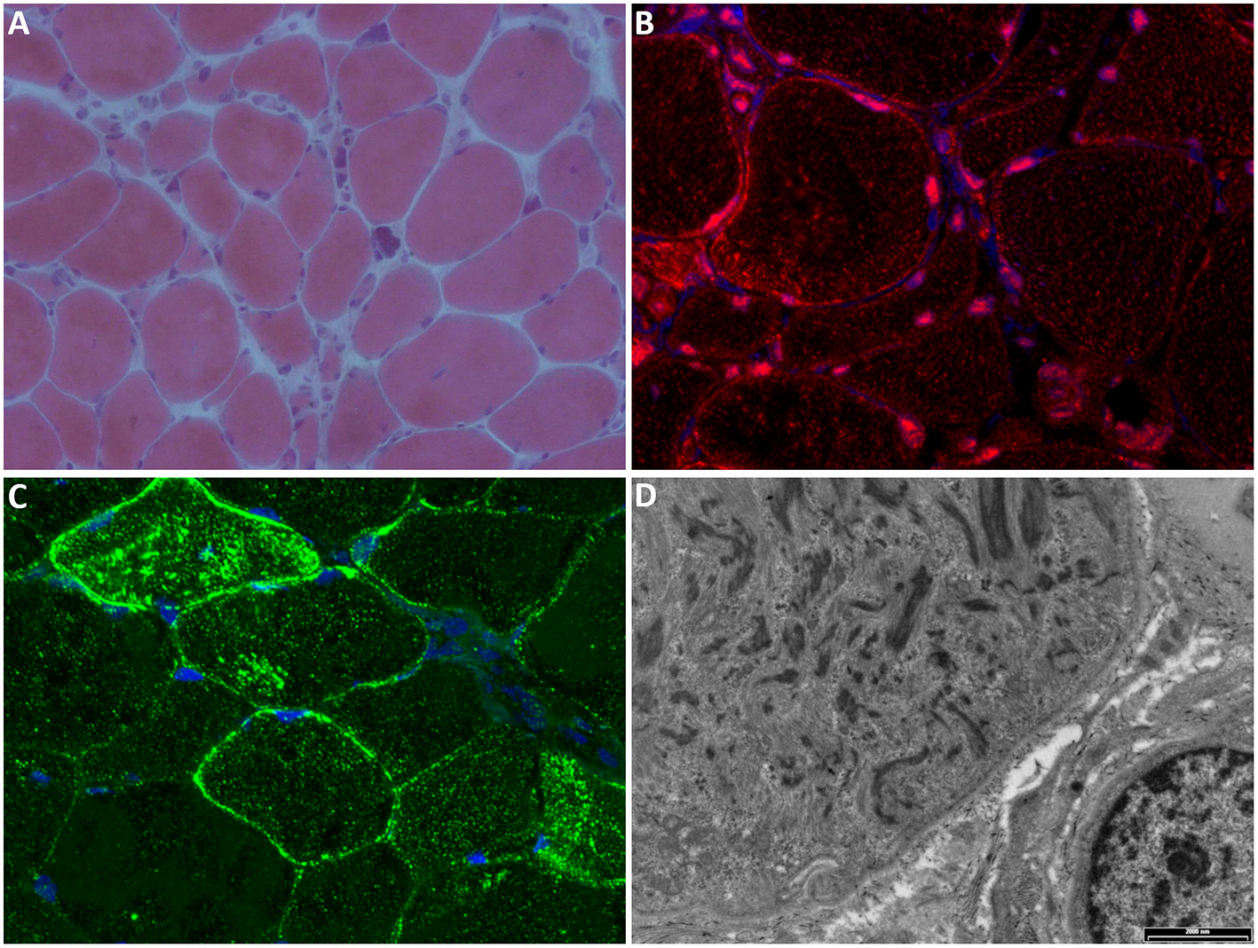
Muscle biopsy. **(A)** Hematoxylin-eosin stain shows marked fiber atrophy, clusters of pyknotic nuclei, and occasional central nuclei (magnification 20×). **(B)** Immunofluorescence (IF) for TNPO3 (red) shows nuclear/perinuclear and cytoplasmic positivity; nuclei are stained with DAPI (blue); magnification 60×. **(C)** IF for SRSF1 (green) shows cytoplasmic and subsarcolemmal aggregates positive for SRSF1; nuclei are stained with DAPI (blue); magnification 60×. **(D)** Electron microscopy showing myofibrillar disarray (scale bar: 2,000 nm).

Two recent papers evaluated the expression of TNPO3 and other proteins normally translocated to the nucleus by TNPO3. The muscles of affected patients showed TNPO3 accumulation in the subsarcolemmal region, in the nuclear/perinuclear area, and also a cytoplasmic signal for TNPO3, likely corresponding to the myofibril array. Among the proteins transported by TNPO3, two proteins, in particular, showed altered expression: RBM4 (RNA binding motif protein 4) seemed to be excluded from the nuclei in patient biopsies (9), while SRSF1 (serine/arginine-rich splicing factor 1) signal appeared reduced in the subsarcolemmal area and occasionally increased, with aggregate formation, in the cytoplasm of several fibers (19). Figure 3 reports the principal features of muscle biopsies from the LGMD D2 patients.

The sporadic cases showed histopathological features similar to the ones described for the Italo-Spanish family, with less severe myopathic changes, and few mitochondrial abnormalities that have been considered as a secondary phenomenon, but a further examination of mitochondrial dynamics might be necessary to elucidate whether TNPO3 has a role in maintaining mitochondrial health (11, 12).

The clinicopathological features of LGMD D2 for both familial and sporadic cases are summarized in Table 1, and even if the muscle biopsies of affected patients show a certain level of variability, they share few main characteristics. In order to explain and to find a connection between these features, it is likely that abnormal sarcomeric protein turnover and assembly are the causes of progressive atrophy and myofiber loss and disarray.

## Differential Diagnosis

The differential diagnosis is based on the interpretation of several clinical data with laboratory and muscle biopsy evaluation. The clinical aspects of this form of LGMD show wide variability, and since with the new diagnostic techniques, such as whole-exome and genome sequencing, *TNPO3* gene variants have been identified in sporadic cases in various countries, sometimes associated with unusual clinical features, the differential diagnosis can be useful for clinicians. The disorders that should be considered in the differential diagnosis of LGMD D2 are:

- Bethlem myopathy (20), due to a mutation of collagen VI, might be present in childhood with finger contractures; other clinical features are joint contractures involving the elbows, ankles, and last four fingers: the “prayer” sign.
- Emery–Dreifuss dystrophy (EDMD) (21) might share overlapping clinical features with LGMD D2 but it has both an X-linked or AD inheritance and usually prominent heart involvement; EDMD is characterized by early contractures at the elbows, Achilles tendon retraction, as well as contractures of neck muscles with the rigidity of the spine.
- Laminopathies result either in a Charcot-Marie-Tooth phenotype or a slowly progressive weakness starting in early childhood with a predominant humeroperoneal distribution.
- Facioscapulohumeral dystrophy (FSHD) (22) and LGMD R1, or calpainopathy (23, 24), might be in differential diagnosis with this LGMD since sometimes both the pelvic and shoulder girdle are involved; however, facial weakness is evident in FSHD, but is minimal in LGMD D2; scapular winging is prominent in calpainopathy and asymmetric in FSHD, and this is a useful diagnostic clue.

Infantile cases present at birth with thin and weak muscles, with the same characteristic features common to several congenital myopathies, but these patients might manifest bulbar symptoms (dysphagia and dysarthria). The differential diagnostic features are from congenital myopathies with hypotonia, such as “central core” or “multicore” myopathies, that have prominent weakness, congenital hip dislocation, while nemaline myopathy shows additional dysmorphic features, such as high arched palate, chest deformity, and an elongated myopathic face. Duchenne boys are noticeably different since they have delayed walking, up to 18 months of age, calf pseudohypertrophy, and high CK. Mental retardation is often present in DMD, congenital myotonic dystrophy, and myotubular myopathy associated with external ophthalmoplegia and facial diplegia. Early-onset LGMD D2 children do not have mental retardation or delayed walking at difference with DMD or congenital myotonic dystrophy. LGMD D2 children might present with diffuse muscle hypotrophy, sometimes twisted feet, and thin leg muscles, but they start walking around 1 year of age, at 3–4 years they do not raise easily from the floor, they have a waddling gait, can climb stairs only using the rail. In their teens, they can walk up to 2–3 km but after 300 m feel fatigued, fall frequently, and use a walker. The most prominent weakness is seen during adolescence, and in a few years, the deambulation becomes slow and is lost around 15 years of age, when a wheelchair is needed.

As far as muscle biopsy changes such as accumulation of cytoskeletal/myofibrillar material is seen in myofibrillar myopathies (25) and LGMD D1 (DMIJB6) (26).

For all patients, laboratory findings (EMG and CK) might be variably present, but they are not conclusive for the diagnosis.

## Genetic Mutations in *TNPO3* That Cause LGMD D2

Limb-girdle muscular dystrophy (LGMD) D2 TNPO3-related is the subtype of dominant LGMDs caused by a mutation in the *TNPO3* gene located on 7q32.1. Several protein-coding and non-coding transcript variants have been found for this gene, the variant NM_012470.4 is the principal one and encodes a protein of 923 amino acids long (NP_036602.1). TNPO3 is expressed in skeletal muscle and many other tissues, and it is a nuclear carrier that shuttles, from the cytoplasm to the nucleus, the serine/arginine-rich proteins (SR proteins). TNPO3 is composed of 20 consecutive hairpin motifs, or HEAT repeats (27), that create a structure with high plasticity responsible for the ability to bind different proteins (28), and that gives TNPO3 a toroidal shape with N- and C-terminal regions facing each other (4). Generally, the N-terminal binds RanGTP, the core region of the protein has the nuclear pore binding site, whereas the C-terminal is the cargo binding domain (29, 30). LGMD D2 was first described in a large Spanish family in 2003, but only in 2013, the sequencing of the whole exome of 4 patients of the original Spanish family lead to the identification of one new variant, shared by all affected subjects, which mapped at 7q32. Thanks to NGS, a single heterozygous nucleotide deletion in the termination codon of *TNPO3* was identified; this mutation results in a frameshift (NM_012470.3: c.2771delA p.^*^924Cext^*^15 in exon 22) that extends the protein by 15 additional amino acids (7, 8). Simultaneously, a heterozygous missense variant [NM_012470.3: c.2453G>A p.(Arg818Gln)] in *TNPO3* was reported in a patient with no familial occurrence of neuromuscular diseases and no other mutations in genes associated with muscular disorders (7, 11). In this sporadic patient with LGMD D2, the heterozygous point mutation changes the arginine in position 818 to glutamine in a highly conserved residue, predicted to be damaging by bioinformatics tools (11).

The Hungarian family carries a heterozygous frameshift variant [c.2767delC p. (Arg923AspfsTer17)] in *TNPO3*. This new pathogenic variant is a frameshift single base pair deletion that results in the extension of the C-terminal domain of the TNPO3 protein. This variant is located in the codon immediately preceding, but it results in a nearly identical longer protein (10).

The third family of LGMD D2, identified in Sweden, showed a new heterozygous mutation (c.2757delC) which causes a frameshift and transfers the stop codon (p. R920Gfs^*^20). The expected outcome is nearly identical to the original *TNPO3* mutation (c.2771delA p.^*^924Cext ^*^15) with an extension in the protein length of 15 amino acids (9).

**Figure 4:**
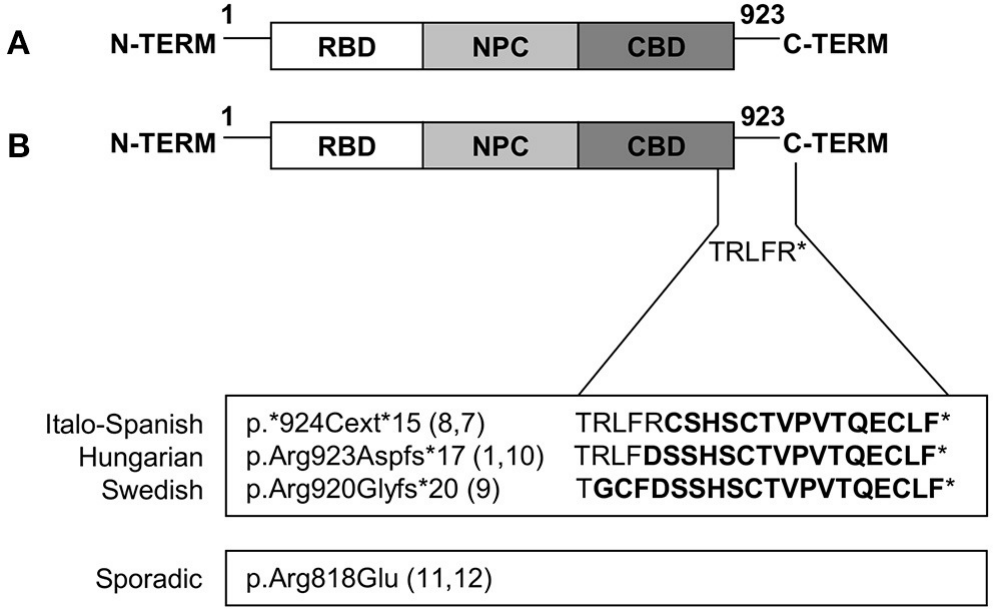
Mutations in *TNPO3* that cause LGMD D2 TNPO3-related. **(A)** Graphic representation of wild-type TNPO3 showing the N-terminal RanGTP-binding domain (RBD), nuclear pore complex (NPC)-binding domain, and a C-terminal cargo-binding domain (CBD). **(B)** Graphic representation of all the described mutations (familial and sporadic cases).

All the different mutations identified so far are summarized in Figure 4.

### Genotype-Phenotype Correlations

Except for the missense mutation described in the sporadic case, all the other variants cause the protein to extend its C-terminal domain, giving rise to a protein that is longer than the wild-type. The amino acid sequence is almost the same, with only slight differences (5 amino acids), thus suggesting that the pathological mechanisms could be widely shared. Genotype-phenotype correlations are complicated by the fact that the identical mutation occurs in various family members, presenting both the childhood and late-onset phenotypes, as well as in paucisymptomatic individuals, showing a variable penetrance. In the Hungarian and Swedish families, the genetic defect seems to produce an early and progressive clinical phenotype, with a different disease progression in time according to the age of the patient. These clinical observations suggest the participation of various other modifiers and environmental features that affect the gene product function, contributing to the high clinical variability.

All the mutations described in this familial LGMD are heterozygous. In muscle, both the wild-type and the mutant form of TNPO3 coexist, but the mutant form seems to have a dominant effect that alters TNPO3's function as a transporter. The pathological mechanism could be either a loss-of-function, with a protein that is unable to transport SR proteins to the nucleus or cause haploinsufficiency.

The missense mutations found in sporadic cases result in a reduced quantity of TNPO3 transcript and protein and do not affect protein localization. This behavior is likely due to messenger instability with a consequent reduction of the protein. The mutation changes an amino acid at position 818, a highly conserved residue whose role is as yet undefined (11). The overall efficiency of transportin-3 seems insufficient to mediate the normal nuclear translocation of its ligands.

This broad scenario of variable phenotypes due to different *TNPO3* mutations with similar effects on the mutant protein raises the possibility that other biological actors or epigenetic modifiers participate in the pathogenetic mechanism.

## Pathogenetic Mechanisms and Clinical Relationships

Torella et al. (7) first described a different mutation in *TNPO3* to the only genetic variant in a sporadic patient with clinical signs of LGMD and no family history of neuromuscular diseases. More recently, and thanks to the addition of *TNPO3* to the panel of genes linked to LGMDs, other families and sporadic cases of LGMD D2 have been described. Despite this, the role of TNPO3 in skeletal muscle and the pathogenic mechanism of the disease are still the open issues.

The identification of the exact pathological mechanism appears challenging for both the limited number of affected patients studied and the high variability in clinicopathological features that characterize this disease (1, 5, 7–11, 14, 18), and the differences in the pattern of expression of some proteins, such as the nuclear/perinuclear localization of TNPO3 (9, 19). The identification of different mutations in *TNPO3*, which seem to have a similar effect at the protein level, may suggest that the pathogenic mechanism is largely shared among families, but how the mutated TNPO3 causes the described variability at clinical and histopathological levels is still unknown.

Transportin-3 normally shuttles, from the cytoplasm to the nucleus, the SR proteins which are involved in RNA metabolism and alternative splicing, thus influencing protein synthesis and turnover in skeletal muscle. Several SR proteins are transported by TNPO3: SRSF1 (splicing factors such as serine/arginine-rich splicing factor 1), SRSF2 (SR-rich splicing factor 2, also known as SC35), RBM4 (RNA binding motif protein 4), and CPSF6 (cleavage and polyadenylation-specific factor 6) (31, 32). The identification of mutations in a protein involved in cytoplasm-to-nucleus transport of SR proteins changes the pathogenic perspective: alteration in translocation and, as a consequence, the function of proteins that take part in alternative splicing regulation might affect protein synthesis as well.

### LGMD D2: A Nuclear Envelopathy With Variable Clinical Expression?

We speculate that different hypotheses can be made about the pathological mechanism of this LGMD such that the mutated TNPO3 is no longer able to bind its cargoes or is not able to transport them to the nucleus correctly, thus altering their physiological functions. The first hypothesis is supported by the fact that the C-terminal domain, which is the one altered by the gene mutation, is the cargo binding domain (33). It is known that TNPO3 interacts with nucleoporins (Nups) at the nuclear pore complex (NPC) through its core region to facilitate the translocation of importin–cargo complexes (31, 34); in a recent experimental study, wild-type TNPO3 seemed to localize to the nuclear envelope, in particular to annulate lamellae (9). The effect of TNPO3 mutation on NPC functions remains to be established, and the described accumulation of TNPO3 in the perinuclear/subsarcolemmal area suggests that normal nuclear functions are damaged in several ways, as confirmed by some nuclear abnormalities described in patient biopsies (8, 9, 18). One might consider LGMD D2 as a nuclear envelopathy, besides laminopathy and emerinopathy. Nuclear positioning in the cells is important for multiple cellular activities during development, immune response, tissue homeostasis, and regeneration. The nucleus is connected to the cytoskeleton by the nuclear envelope proteins and the NPC. However, LGMD D2 has some specific elements since skeletal muscle is almost exclusively affected, with no cardiac involvement, differently from what has been observed in both laminopathy and emerinopathy.

### LGMD D2: Altered Nuclear Transport at the Base of Abnormalities in Protein Turnover and Myofibrils Synthesis?

The immunohistochemical and ultrastructural studies showed accumulation of myofibrillar proteins and myofibrillar disarray, suggesting that it is a myofibrillar-like myopathy (14, 18). These findings, in addition to the autophagic reaction revealed by the formation of autophagosomes, p62 expression, and the upregulation of MURF-1, lead us to suppose that autophagy might have a role in myofiber atrophy and is associated with the progression of the disorder (7, 14). Autophagic block seems to play a role in the progression of the disease, revealed by protein aggregates and damaged mitochondria. P62 plays a significant role in the autophagy of ubiquitinated protein aggregates, transporting them within the autophagy-lysosome degradation system. Moreover, the nuclear alterations observed in muscle biopsies may be due to the inability of mutated TNPO3 to migrate the SR proteins through the nuclear membrane, interfering or blocking their activity in alternative splicing and altering protein synthesis and myofibrillar assembly. The SR proteins, which normally work as essential splicing factors, influence posttranscriptional gene regulation, affecting the proteomic diversity in muscle and contributing to the formation and maintenance of skeletal muscle (35, 36). An altered protein turnover is clearly linked to the activation of the autophagic machinery and probably leads to fiber atrophy. The involvement of TNPO3 in muscle differentiation has been reported in a study on C2C12 myoblasts, showing the variation of TNPO3 in the nuclear and cytoplasmic compartments in myoblasts that are differentiating into myotubes. These data suggest a possible involvement of TNPO3 and SRSF1 in a dynamic interaction that follows the myogenic process and could influence the proteomic network during myogenesis (37).

### Abnormal Dimerization of TNPO3

Another hypothesis on the pathogenesis of LGMD D2 comes from the possibility that the heterozygous mutation has a dominant-negative expression. Investigation of mRNA from patients showed the coexistence of both wild-type and mutant transcripts in similar amounts (8), allowing the presence, at least partially, of normal protein with normal nuclear transport. TNPO3 carries out nuclear import functions (4, 38), and these observations might confirm that TNPO3 acts as a dimer when shuttling its cargoes to the nucleus. Our hypothesis is supported by a recent study on C2C12 myoblasts, showing that TNPO3, once in the nucleus, had a globular volume that was bigger than in the cytoplasm, suggesting the formation of dimers (37). Since TNPO3 works as a dimer, it is likely that the mutant protein fails in the formation of functional dimers or alters the activity of the dimers themselves, preventing the correct nuclear transport of SR proteins and participating in the alterations previously described. Additional functional studies are needed to find the real mechanism responsible for this disease.

## Future Perspectives

### From *TNPO3* Mutation to HIV Resistance

The LGMD D2 pathogenetic cascade is of interest both to scientists involved in neuromuscular disorders and to those dealing with HIV infection. It is well-known that TNPO3 directly participates in nuclear import and infection of HIV-1 (30, 38, 39), but it is also probable that this neuromuscular disease makes affected subjects immune to HIV infection, holding promise for research in this field. In a recent study, Rodriguez-Mora et al. observed that the CD4 lymphocytes, isolated from patients with LGMD D2 with the mutation in *TNPO3* described in the Italo-Spanish family, are resistant to HIV-1 infection *in vitro* (40). So, we are faced with an *in vivo* situation in which the genetic defect that causes this neuromuscular disease also gives resistance to HIV infection. The *TNPO3* mutation causing LGMD D2 may represent a natural model for understanding the pathogenesis of both diseases and for designing new drugs for the treatment of both diseases.

### microRNA as Disease Biomarkers

Circulating muscle-specific microRNAs (myomiRNAs) are released in the bloodstream by muscles in response to physiological or pathological processes, and they could serve as cell signaling molecules, mediating cell-to-cell communication, regarding muscle repair, regeneration, and remodeling (41–43). It has been observed that myomiRNA dysregulation occurs first in muscle biopsy and later extends to the plasma, suggesting a spill-over mechanism that might be of interest when considering a possible role as a biomarker (44). MyomiRNA expression has been shown to vary in the presence of muscle disease, suggesting that they could be used as available biomarkers (45, 46). It is important to choose appropriate biomarkers to monitor, evaluate, and investigate the evolution of disorders with clinical heterogeneity, such as LGMD D2. A recent study demonstrated that the expression of miR-206 was highly increased in patients affected by LGMD D2 and this increase seemed to correlate with patient disease severity and progression when compared to other slow evolving patients and controls (47). The observed increase in expression of miR-206, which is known to be directly involved in the regulation of muscle differentiation (41), supports the hypothesis that alterations in myogenesis contribute to the pathogenesis of this disease, as shown by type I fiber uniformity (14).

### New Research Strategies

The CRISPR/Cas9 technique opens a new perspective on the creation of disease models and future therapeutic strategies. Genome editing through CRISPR/Cas9 helps to realize stable cell lines carrying the TNPO3 mutation to be used as disease models *in vitro*. A cellular model of the disease helps to study the exact pathogenic mechanism and could be used for specific drug testing as well. CRISPR/Cas9 could be used to correct the TNPO3 mutation in myoblasts isolated from patients, giving proof of concept for a possible personalized molecular therapy approach.

Similarly, animal models of the disease, for example, using fly (*Drosophila*) (48) or zebrafish (*Danio rerio*), will be useful in functional studies to understand the pathogenesis of this disease and will provide additional tools for drug screening that could later be applied in the clinical treatment of patients.

## Conclusion

Limb-girdle muscular dystrophy (LGMD) is extremely challenging to diagnose early, yet prompt diagnosis is important, not only in terms of genetic counseling but also for the prognosis and clinical management of the patients. There is some disagreement about how to define the LGMD D2 phenotype, which was usually done on clinical grounds, the characteristics, namely, severe weakness and marked muscle atrophy. The use of muscle MRI to systematically analyze single muscles needs to be studied further, but it appears to be a very good tool for evaluating muscle atrophy in this LGMD. The accurate diagnosis of LGMD D2 is made more easily with NGS techniques and by the introduction of *TNPO3* in the panels of muscular disease-related genes. Recently, the analysis of myomiRNAs, in particular miR-206, has been proposed as a promising biomarker for the clinical follow-up of LGMD D2 (47). Such a non-invasive methodology will be used in the future in evaluating the effects of various types of treatments using quantitative indices in follow-up and clinical outcomes.

We review the clinical, genetic, and histopathological characteristics of LGMD D2, and hypothesize three main pathogenetic mechanisms of the disease. The pathogenetic hypotheses are all attributable to the defective role of TNPO3 in transporting the SR proteins involved in alternative splicing and, as a consequence, altering protein synthesis and myofibrillar assembly. Dysregulated protein synthesis is strictly related to the development of myofibrillar protein aggregates and the activation of degradation mechanisms. This causes significant muscle weakness and damage by producing a negative balance in protein turnover, which is highlighted by the expression of autophagic markers as observed in muscle atrophy. We are planning further functional studies to investigate and identify the pathomechanisms that will be followed by drug therapy. So far, the treatment of affected cases is only symptomatic and necessitates future personalized molecular advances.

## Author Contributions

RC, MTR, and CA wrote the manuscript. SP, CA, and GC reviewed the manuscript. RC, MTR, CA, and GC designed the general outline for the manuscript. All authors contributed to the article and approved the submitted version.

## Funding

This project has received funding from AFM-Telethon (Reference number 22392).

## Conflict of Interest

The authors declare that the research was conducted in the absence of any commercial or financial relationships that could be construed as a potential conflict of interest.

## Publisher's Note

All claims expressed in this article are solely those of the authors and do not necessarily represent those of their affiliated organizations, or those of the publisher, the editors and the reviewers. Any product that may be evaluated in this article, or claim that may be made by its manufacturer, is not guaranteed or endorsed by the publisher.
